# Autorefraction Pitfalls in Extended Depth-of-Focus Pseudophakia: A Case Report

**DOI:** 10.7759/cureus.87409

**Published:** 2025-07-07

**Authors:** Andreas F Borkenstein, Eva-Maria Borkenstein

**Affiliations:** 1 Ophthalmology, Borkenstein and Borkenstein Private Practice, Privatklinik der Kreuzschwestern Graz, Graz, AUT; 2 Ophthalmology, Borkenstein and Borkenstein Research and Laboratory GmbH, Graz, AUT

**Keywords:** autorefraction error, edof iol, interdisciplinary care, pseudophakia, spectacle overcorrection, subjective refraction

## Abstract

Extended depth-of-focus (EDOF) intraocular lenses (IOLs) are increasingly used to provide a broad range of vision after cataract surgery. However, their unique optical design can produce misleading results when postoperative refraction is assessed using automated methods. We report three pseudophakic patients implanted bilaterally with Tecnis PureSee IOLs, all of whom demonstrated excellent uncorrected visual acuity (1.0) but appeared myopic on autorefractor testing. Based on these values, opticians prescribed minus-powered spectacles, including night-driving glasses and progressive lenses. All three patients developed asthenopic symptoms such as headaches, nausea, diplopia, or dizziness. Symptoms resolved rapidly upon discontinuation of spectacles. However, diagnosis was delayed by 2.5 to 4 weeks. These cases illustrate the risk of overcorrection due to objective refraction artifacts in EDOF optics. The myopic shift observed on autorefraction did not reflect functional visual performance. Lack of communication between surgeons and opticians, as well as unawareness of IOL model-specific refraction behavior, contributed to incorrect prescriptions and patient dissatisfaction. Subjective refraction and functional assessment are essential following EDOF IOL implantation. Communication between ophthalmologists and optical professionals should be improved, and IOL-specific refractive behavior must be considered to avoid unnecessary spectacle correction and ensure optimal visual outcomes with high patient satisfaction. Understanding the optical behavior of new IOLs and tailoring refractive care accordingly should be standard practice in the management of premium IOL patients.

## Introduction

In recent years, intraocular lens (IOL) technologies have evolved beyond the restoration of distance visual acuity after cataract surgery. Modern designs increasingly target functional vision at various distances, enhanced contrast sensitivity, and reduced reliance on spectacles. This shift has led to the development and widespread use of so-called “premium” IOLs. These lenses address diverse visual needs and have redefined postoperative expectations for both patients and clinicians.

Extended depth-of-focus (EDOF) IOLs are increasingly used in cataract surgery to improve functional vision across distance and intermediate ranges while minimizing dysphotopsia. Their non-diffractive optical design aims to preserve contrast sensitivity and reduce photic phenomena compared to traditional multifocal lenses [1-3].

Postoperative refraction is routinely assessed using automated refractometers in both surgical and community optometric settings. While efficient and widely available, autorefraction may produce misleading results in eyes implanted with modern IOL optics, particularly EDOF designs [4-6].

A characteristic phenomenon is the appearance of a myopic shift, despite excellent uncorrected distance acuity, resulting in inappropriate spectacle prescriptions when interpreted without awareness of lens-specific behavior [6,7].

This case report presents three patients implanted with Tecnis PureSee EDOF lenses who experienced asthenopic symptoms due to overcorrection based on autorefraction alone. These cases highlight the importance of subjective verification, interdisciplinary communication, and awareness of the limitations of wavefront-based measurement techniques in pseudophakic eyes.

## Case presentation

Case 1

A 64-year-old woman with bilateral cataracta senilis underwent an uneventful bilateral cataract extraction with implantation of Tecnis PureSee IOLs, aiming for emmetropia in the right eye (0.00 sph) and slight myopia (-0.25 sph) in the left eye. At the one-week postoperative follow-up, uncorrected distance visual acuity (UCDVA) was 0.9 (decimal) in both eyes and 1.0 (decimal) binocular, and binocular near visual acuity (UCNVA) reached 0.7 (decimal) using a reading distance of 50 cm, without need for correction. The patient reported high visual satisfaction at this stage. However, postoperative autorefraction measurement showed -1.00 D/-0.25 D cyl at 100° OD and -1.25 D/-0.25 D cyl at 90° OS. Based on these readings, the optician recommended a distance prescription for UV-blocking sunglasses and clear driving glasses intended for nighttime driving or bad weather conditions. After wearing those glasses, the patient developed continuous headaches, nausea, and ocular burning, which persisted over several hours. Symptoms resolved rapidly after discontinuation of spectacle use. The functional mismatch between objective measurements and the patient’s subjective visual experience was identified only after this episode.

Case 2

A 78-year-old man underwent uncomplicated bilateral cataract surgery with implantation of Tecnis PureSee IOLs, targeting emmetropia in both eyes (0.00 sph OU). At the one-week postoperative follow-up, UCDVA was 1.0 (decimal) in each eye and 1.0 (decimal) binocularly. UCNVA reached 0.8 (decimal) at a reading distance of 55 cm, and no correction was required. The patient reported high subjective satisfaction and no visual complaints. At a follow-up visit with his optician, postoperative autorefraction measured -1.00 D/-0.50 D cyl at 20° OD and -1.00 D/0.00 D cyl OS. Based on these findings, the optician recommended prescription spectacles for driving and attending theater performances. Shortly after beginning to use the glasses, the patient developed significant dizziness, ocular discomfort, and visual confusion, particularly under dim lighting conditions such as the theater. These symptoms subsided promptly following discontinuation of the spectacles. The mismatch between objective measurements and the patient’s actual visual experience led to a delayed recognition of the problem. The patient expressed frustration regarding unnecessary spectacle costs and multiple follow-up visits, despite an otherwise successful surgical outcome.

Case 3

A 67-year-old woman with bilateral cataracts and moderate pre-existing myopia (-4.50 D sph) underwent uneventful cataract surgery with implantation of Tecnis PureSee IOLs. The surgical target was a slight residual myopia of -0.50 D in both eyes to support near function. At the one-week postoperative follow-up, UCDVA was 0.9 (decimal) in both eyes and 1.0 (decimal) binocularly. UCNVA reached 0.8 (decimal) at a reading distance of 40 cm, and no optical correction was required at this time. The patient reported high visual satisfaction in daily activities. Two weeks later, at a routine visit with her optician, postoperative autorefraction showed -1.50 D in the right eye and -1.25 D in the left eye. Based on these measurements, she was prescribed two pairs of spectacles: one for distance vision (e.g., nighttime driving) and one progressive addition lens for near work and household tasks. Soon after initiating spectacle use, the patient developed persistent frontal headaches, instability while walking, and transient diplopia during gaze shifts. These symptoms improved markedly after she stopped wearing the glasses. The underlying issue, overcorrection based on autorefractor readings in an otherwise functionally emmetropic patient, was identified three weeks later. She expressed clear dissatisfaction due to the unnecessary financial burden of approximately €900 spent on the spectacle corrections (Table 1).

**Table 1:** A summary of the three cases. An overview of visual acuity outcomes, autorefraction findings, prescribed spectacle types, and associated symptoms in three patients with Tecnis PureSee EDOF IOLs. The table highlights the mismatch between objective measurements and functional vision. UCDVA: uncorrected distance visual acuity; UCNVA: binocular near visual acuity; EDOF: extended depth-of-focus; IOL: intraocular lens; OD: right eye; OS: left eye; OU: both eyes

Case	Age (years)/Sex	IOL type/Target refraction	UCDVA (binocular, decimal)	UCNVA (binocular, decimal at distance in cm)	Autorefraction	Prescribed spectacle type	Symptoms
1	64/F	Tecnis PureSee/+-0.00 OD and -0.25 OS	1.0	0.7 at 50 cm	-1.00/-0.25 at 100° OD and -1.25/-0.25 at 90° OS	Sunglasses + driving glasses (night/bad weather)	Headache, nausea, ocular burning
2	78/M	Tecnis PureSee/+-0.00 OU	1.0	0.8 at 55 cm	-1.00/-0.50 at 20° OD and -1.00/0.00 OS	Driving glasses + theatre use	Dizziness, discomfort, visual confusion
3	67/F	Tecnis PureSee/-0.50 OU	1.0	0.8 at 40cm	-1.50/0.00 OD and -1.25/0.00 OS	Driving glasses + progressive lens for near/household tasks	Headache, instability, diplopia

The data reinforce our interpretation that the observed myopic shift on autorefraction was artifactual rather than anatomical or surgical (Table 2).

**Table 2:** Preoperative biometric data for all three cases. IOL: intraocular lens; OD: right eye; OS: left eye; OU: both eyes

Case	Eye	Keratometry K1 (D)	Keratometry K2 (D)	Anterior chamber depth (mm)	Axial length (mm)	Implanted IOL power (D)	Target refraction (D)
Case 1	OD	42.71	43.11	3.2	23.84	20.5	0
Case 1	OS	42.61	42.89	3.6	24.73	18.0	-0.25
Case 2	OD	41.98	42.43	3.1	24.65	18.5	0
Case 2	OS	42.43	42.76	3.9	25.12	17.0	0
Case 3	OD	42.98	43.32	4.0	26.8	12.0	-0.50
Case 3	OS	42.78	43.01	4.2	25.9	15.0	-0.50

## Discussion

The presented cases underscore the limitations of objective refraction when applied to eyes implanted with advanced (“premium”) IOLs. All three patients demonstrated excellent UCDVA yet received myopic spectacle prescriptions based solely on autorefractor data. This mismatch between functional vision and automated measurements reflects the interaction between modern IOL optics and infrared-based metrology.

According to the European Society of Cataract and Refractive Surgeons (ESCRS), premium IOLs can be grouped by optical mechanism and intended visual performance into several categories [1]. Enhanced monofocal IOLs maintain a monofocal profile but incorporate asphericity or refractive transitions to improve intermediate vision (e.g., Tecnis Eyhance ICB00) [2]. EDOF IOLs provide a continuous range from far to intermediate, using diffractive or non-diffractive wavefront-shaping elements (e.g., Tecnis PureSee DEN00V and Tecnis Odyssey DRN00V) [3,4]. Multifocal or hybrid IOLs (e.g., Tecnis Synergy DFR00V) extend focus further into the near range by combining multiple focal planes [5,6]. While non-diffractive EDOF lenses preserve image quality and reduce glare, they can interfere with standard measurement techniques. In particular, a falsely myopic result is often observed on autorefraction, even in the presence of excellent visual function [6-8]. This phenomenon is likely multifactorial (Table 3).

**Table 3:** Summary of optical and measurement-related mechanisms contributing to falsely myopic autorefraction in eyes with EDOF IOLs. IR: infrared; EDOF: extended depth-of-focus; IOL: intraocular lens

Mechanism	Category	Explanation	Clinical impact
IR focal shift	Measurement-related	Infrared light focuses behind the visible light plane due to its longer wavelength	Devices misjudge the focal point, creating an apparent myopic error
Wavefront-based interpretation	Device limitation	Autorefractors interpret the return wavefront shape; steeper wavefronts are read as increased negative power	Systematic overestimation of myopia, especially in pseudophakic eyes
Chromatic dispersion	Material-related	Hydrophobic acrylic IOLs with low Abbe numbers cause wavelength-dependent focal shifts	Amplifies the discrepancy between the IR and visible spectrum, increasing false myopia
Central optical modulation	Optical	EDOF IOLs reshape the central optical zone to extend the depth of focus	Alters the refractive profile, often misinterpreted by autorefractors
Depth of focus tolerance	Optical	EDOF lenses provide broad defocus tolerance, masking small residual errors	Patients maintain good vision despite objective error, leading to unnecessary prescriptions

First, EDOF lenses modulate the central wavefront to stretch the depth of focus, which can alter how automated devices detect the return image. Second, autorefractors typically use infrared (IR) light (800-900 nm) to assess refractive error. Although IR light focuses posterior to the retina (suggesting a hyperopic displacement), the returning wavefront is steeper and interpreted by the device as increased negative power. Autorefractors do not assess the focal point directly but infer refractive status from wavefront curvature. This wavefront-based analysis leads to a systematic overestimation of myopia in pseudophakic eyes with EDOF optics [7-9].

Moreover, the chromatic dispersion of IOL materials, especially hydrophobic acrylics with low Abbe numbers, enhances wavelength-dependent focal differences, contributing further to IR-visible mismatch [8].

Clinically, this artifact may go unnoticed unless the patient reports symptoms. All three of our patients were functionally emmetropic, yet developed headaches, dizziness, or diplopia after using minus lenses prescribed based on autorefraction alone (“overcorrection”). In all cases, symptoms resolved after stopping spectacle use. To confirm that the observed myopic readings were not attributable to biometric error or incorrect IOL selection, we presented key preoperative biometric data in Table 2. This includes axial length, corneal power (K1/K2), anterior chamber depth, and implanted IOL power with target refraction. The calculations were performed using the ESCRS calculation program, and at least two formulas (e.g., Barrett and Kane) were used as cross-checks. Postoperative subjective refraction further supports that no significant residual refractive error was present.

The biometric data and the good uncorrected visual acuity support the assumption that true refractive error was minimal and not due to surgical miscalculation, consistent with previous findings on biometry accuracy in EDOF implantation [10].

From a systems perspective, these cases expose a communication gap between cataract surgeons and community opticians. Without access to IOL model information or awareness of its optical profile, opticians may unknowingly overcorrect eyes that functionally do not require it. Implementing IOL identification cards, digital records, or structured communication protocols could help reduce such errors. Patients often maintain good visual performance across a range of defocus values, even with small residual refractive errors, as shown in prior studies on EDOF lens performance [11-13]. Clinicians should interpret postoperative refraction in the context of subjective patient feedback and neuroadaptation. In patients with EDOF lenses and satisfactory visual function, delaying spectacle prescription is often the safest course, especially when automated refraction suggests minor myopia without clinical symptoms [9,14].

Overall, successful postoperative care of premium IOL patients depends not only on surgical precision but also on interdisciplinary coordination and refractive awareness across all care providers involved.

## Conclusions

EDOF IOLs provide excellent functional visual outcomes, particularly for distance and intermediate vision. However, their unique optical design may lead to misleading postoperative measurements when standard autorefractors are used. In all cases presented, patients demonstrated functionally emmetropic outcomes with excellent uncorrected visual acuity, yet were mistakenly overcorrected based on device-generated myopic readings. These cases underscore a critical gap between objective refraction and real-world visual function in pseudophakic eyes with EDOF optics. Importantly, symptoms such as asthenopia and visual discomfort resolved only after discontinuation of the unnecessary spectacles. To optimize outcomes and avoid patient dissatisfaction, a refined postoperative approach is essential. This includes delaying spectacle prescriptions until subjective visual stability is confirmed, prioritizing patient-reported visual quality over automated metrics, and fostering consistent communication between surgeons, refractionists, and optical professionals. Understanding the optical behavior of EDOF lenses and tailoring refractive care accordingly should be standard practice in the management of premium IOL patients.
